# A First Step Toward Unraveling the Energy Metabolism in Endurance Horses: Comparison of Plasma Nuclear Magnetic Resonance Metabolomic Profiles Before and After Different Endurance Race Distances

**DOI:** 10.3389/fmolb.2019.00045

**Published:** 2019-06-12

**Authors:** Laurence Le Moyec, Céline Robert, Mohamed N. Triba, Nadia Bouchemal, Núria Mach, Julie Rivière, Emmanuelle Zalachas-Rebours, Eric Barrey

**Affiliations:** ^1^UBIAE EA 7362, Université Evry, Université Paris-Saclay, Évry, France; ^2^Animal Genetics and Integrative Biology (GABI - UMR1313), INRA, AgroParisTech, Université Paris-Saclay, Jouy-en-Josas, France; ^3^École Nationale Vétérinaire d'Alfort, Maisons-Alfort, France; ^4^CSPBAT, UMR 7244, CNRS, Université Paris 13, Sorbonne Paris Cité, Bobigny, France

**Keywords:** metabolomics, biochemistry, energetics, exercise physiology, endurance, NMR, long exercise, horse

## Abstract

Endurance racing places high demands on energy metabolism pathways. Metabolomics can be used to investigate biochemical responses to endurance exercise in humans, laboratory animals, and horses. Although endurance horses have previously been assessed in the field (i.e., during races) using broad-window Nuclear Magnetic Resonance metabolomics, these studies included several different race locations, race distances, age classes, and race statuses (finisher or elimination). The present NMR metabolomics study focused on 40 endurance horses racing in three race categories over 90, 120, or 160 km. The three races took place in the same location. Given that energy metabolism is closely related to exercise intensity and duration (and therefore distance covered), the study's objective was to determine whether the metabolic pathways recruited during the race varied as a function of the total ride distance. For each horse, a plasma sample was collected the day before the race, and another was collected at the end of the race. Sixteen, 15, and 9 horses raced over 90, 120, and 160 km, respectively. Proton NMR spectra (500 MHz) were acquired for these 80 plasma samples. After processing, the spectra were divided into bins representing the NMR variables and then classified using orthogonal projection on latent structure models supervised by the sampling time (pre- or post-race) or the distance covered. The models revealed that the post-race metabolomic profiles are associated to the total ride distance groups. By combining biochemical assay results and NMR data in multiblock models, we further showed that enzymatic activities and metabolites are significantly associated to the race category. In the highest race category (160 km), there appears to be a metabolic switch from carbohydrate consumption to lipid consumption in order to maintain glycaemia. Furthermore, signs of protein breakdown were more apparent in the longest race category. The metabolic shift seen in the different racing categories could be related to a mixture of three important factors that are the ride distance, the training status and the inherited endurance capacity of the various horses competing.

## Introduction

The positive or negative effects of exercise on metabolic pathways have been widely studied in humans and animal models. In this field, untargeted multiparameter studies have emphasized the ability of metabolomics to highlight the metabolic pathways involved in exercise (Pechlivanis et al., 2013; Bassini and Cameron, 2014). Metabolomics has been used to investigate adaptive metabolism responses to endurance exercise, such as marathon running in humans (Lewis et al., 2010) and treadmill exercise to exhaustion in mice and rats (Le Moyec et al., 2012; Monleon et al., 2014). Endurance horses have also been investigated in the field (i.e., during real endurance races) using NMR-based metabolomics (Le Moyec et al., 2014; Luck et al., 2015). Equine athletes start training for endurance races at the age of 4, and tend to compete until the age of 15 or so. The total distance covered is split into 30–40 km loops, with a veterinary control (“vet gate”) at the end of each loop. The veterinarians ensure that horses are not suffering from lameness or dehydration, and that their heart rate is low enough to start another loop. This process is repeated until the horses have covered 90, 120, or 160 km. Consequently, around 50% of the starters tend to be eliminated because of fatigue, lack of fitness, or another physiological or biomechanical disorder. Hence, the challenge for a trainer is to enable his/her horse to run as fast as possible while maintaining good health and rapid cardiac recovery. At the end of the race, a veterinarian checks the horse again and designates it as a finisher or a non-finisher (i.e., elimination).

Endurance racing places high physiological demands on energy metabolism pathways. Although horses have previously been assessed in the field (i.e., during endurance races) with NMR metabolomics, these studies included several different race locations, race distances, age classes, and race statuses (finisher or elimination). Despite the marked heterogeneity of the study populations, a comparison of plasma taken before and after an endurance race showed that the metabolic profile depends on the characteristics of the exercise (Le Moyec et al., 2014). A subsequent study showed that 6-year-old horses in a 90 km race had a more glycolytic metabolic profile than older horses in a longer (160 km) race (Luck et al., 2015). However, these studies did not examine the difference between effects of age or race category (i.e., total ride distance). In the present study, we had the opportunity to standardize the environmental conditions (the race location, weather conditions and ground surface) and thus focus on changes in energy metabolism in relation to the race categories (i.e., total ride distance of 90, 120, or 160 km). Given that energy metabolism is closely related to the intensity and duration of exercise (and therefore the race distance), the primary objective of the present investigation was to determine the plasma metabolic profile shift at the completion of three different racing distances (90, 120, and 160 km).

## Materials and Methods

### Inclusion Criteria and Sample Collection

Blood samples were collected from horses racing over 90, 120, or 160 km at an endurance competition in Fontainebleau (France) on October 16th (in the afternoon before the event) and October 17th (at the end of the event) 2015. All the horses had been road-transported to arrive in Fontainebleau between 4 and 36 h before sampling. A total of 52 horses (out of 192) were initially included in the study following the provision of informed consent by the owners and riders. The study protocol had been reviewed and approved (as part of the GenEndurance project) by the local animal care and use committee (ComEth EnvA-Upec-ANSES; reference: 11-0041, dated July 12th 2011). Venous blood was drawn from the jugular vein into dry, EDTA and lithium heparin tubes for biochemical assays and fluoride-oxalate tubes for metabolomics analyses. Serum and plasma samples were maintained at +5°C until analysis.

The day of the race, the weather was cool and cloudy, with a temperature between 6 and 13°C, a total of 2 mm of precipitation and a humidity between 90 and 100%. Each rider was free to manage his horse according to his habits. For another part of the study, we collected data on the horses' diet before and during the race. There are of course individual variations, nevertheless, the management of horses is basically the same: all have the opportunity to drink, eat a small amount of concentrates, and hay between each loop. From the second loop, the riders can stop at predefined crew points (every 10 km) to offer water to their horse. Blood samples were collected during the afternoon the day before the endurance race and between 20 and 30 min after the end of the race, i.e., about 24 h after pre-race sampling: the comparison of pre- and post-race results is therefore not affected by potential circadian variations in metabolism. Similarly, all samples were taken away from food intakes. The time schedule of each race is reported in the Supplemental Table S1. Plasma samples collected for NMR analysis were frozen immediately after centrifugation and stored at −80°C. Forty of the 52 horses finished the race, and so the corresponding 80 samples were included in the present analysis. Sixteen horses raced over 90 km, 15 raced over 120 km, and 9 raced over 160 km.

### Acquisition and Processing of NMR Spectroscopy Data

Plasma samples were thawed at room temperature. In 5 mm NMR tubes, 600 μL of plasma were added to 100 μL deuterium oxide (for field locking). Proton spectra were acquired at 500 MHz and 300 K on a Bruker Avance III spectrometer with a 5 mm reversed QXI Z-gradient high-resolution Bruker probe. One-dimensional Carr-Purcell-Meiboom-Gill (CPMG) free induction decay (FID) acquisition sequence was used. The acquisition methods were described previously (Luck et al., 2015). Shortly, the CPMG sequence was applied using a tau value (T2-Filter) of 31 ms, water signal suppression during the relaxation time and 64 scans. The CPMG sequence is used to suppress the broad signal from proteins and lipids according to their short T2 relaxation time.

Automatic signal processing was performed with an in-house routine code using NMRpipe. The same Fourier transform with a 0.3 Hz line broadening, chemical shift calibration (according to the C1-alpha-glucose doublet at 5.23 ppm), and spectral phasing was performed manually. The processing method also included an integral calculation of 0.001 ppm regions from 9.5 to 0 ppm (referred to as bins). This generated an X matrix, used for multivariate statistical analysis, in which each line is a spectrum (i.e., a sample from a horse) and each column is a bin (labeled with its mean chemical shift).

### Multivariate Analysis

The X matrix (containing NMR data) was normalized according to the spectra using the probabilistic quotient method (Dieterle et al., 2006) and the bins, corresponding to variables in the statistical analysis, were centered and scaled to the unit variance.

The two analytical methods applied were also described previously (Luck et al., 2015): unsupervised principal component analysis (PCA) and the supervised orthogonal projection on latent structure (O-PLS performed using an in-house routine written in MATLAB (The MathWorks, Natick, MA, USA) based on the method described by Trygg and Wold (2002). The PCA was first applied to detect any separation between groups and the O-PLS analysis was performed to identify differences between sample spectra as a function of supervising factors. These factors were coded in a so-called Y matrix, which included the time point (pre- or post-race) and the distance covered by the finisher horses (90, 120, or 160 km). The quality of the O-PLS model was assessed by calculating the R^2^Y fit parameter and the Q^2^Y cross-validated coefficient of determination parameter. Only models achieving Q^2^Y value over 0.5 were considered. A permutation test (*n* = 999 permutations) was performed as another means of internally validating method (Triba et al., 2015). This process also checks for the absence of artifactual over-fitting by the O-PLS models; all those presented here met this condition.

A score plot and a loading plot were computed to illustrate the results of the O-PLS models. The loading plot represents the covariance between the Y-response matrix and the signal intensity of the various spectral domains. Colors were also used in the loading plot, depending on the Pearson's correlation between the corresponding bin intensity and the Y variable. Metabolites were considered to be discriminating when they corresponded to bins with a correlation value of I0.5I or more. In such a case, it was considered that the level or relative amount of the corresponding metabolite was modified according to the supervising factor. The *p*-value corresponding to the null “Ho: no correlation” is given for *R* = l0.5l in each model in **Table 2**. The calculated *p*-values correspond the *p*-values of a Student test between the two groups.

In the present study, we removed inter-individual variability by using the paired method (Westerhuis et al., 2010). Inside this within X matrix (excluding the inter-individual variability), only post-race samples were used to compute an O-PLS model with race distance as the supervising factor.

In order to perform a more integrated analysis, biochemistry and NMR data were considered in an unsupervised multiblock common component and specific weights analysis (CCSWA) (Qannari et al., 2001). This method enables the simultaneous analysis of heterogeneous data from the same samples. Each type of data constitutes a block; in the present study, the blocks X_BIO_ and X_NMR_ corresponded to the biochemical assay and NMR datasets, respectively. The CCSWA is used to identify common components, i.e., a common source of variability in the two blocks. However, each block can make a specific contribution (i.e., a specific weight) to the common component. These specific weights have to be considered when interpreting the model's output. Before building the CCSWA model, the biochemical assay and NMR datasets are scaled so that each block and each variable within a block have the same initial weight. Thus, variables that constituted X_BIO_ and X_NMR_ were scaled to unit variance, and the resulting matrices were scaled so that they had the same variance.

### Biochemical Assays

Plasma samples taken before and after the race were assayed on an RX Imola system for the following analytes: total protein, creatinine, creatine phosphokinase (CK), aspartate amino transferase (ASAT), total bilirubin, gamma glutamyl transferase, serum amyloid A (SAA), beta hydroxybutyrate (BHB), and non-esterified fatty acids (NEFAs).

## Results

### Horses and Race Data

The mean characteristics of the horse's group in the three race categories are presented in Table 1. For each horse, their heart rates measured at each vet-gate and their speeds on each step are shown in the Supplemental Table S2. Groups were not different for horse breeds with mainly purebred Arabian then half-Arabian horses. For age, the three groups were not different on average. Nevertheless, the endurance rules impose a minimum age of 7 years for 120 km and 8 years for 160 km races. There were therefore no horses aged 6 and 6–7 years old, respectively, in these two distance groups. Heart rate and speed data during the same part of the race were not different between the three distance groups (Supplemental Table S1). The average speed of the last step of the race before arrival was measured and summarized for each race distance in Supplemental Table S2. We observed that the horses competing in the 160 km race finished at slower averaged speed (14.44 ± 2.22 km/h) that the two other race distance 90 and 120 km (17.64 ± 2.99 and 17.93 ± 2.99 km/h, respectively).

**Table 1:** Description of the three groups of horses based on the race distance.

	**Race distance**	**90 km**	**120 km**	**160 km**
Horses		16	15	9
Breed	Anglo-Arabian	2	0	1
	Arab	10	11	7
	Half Arab	3	4	1
	Other	1	0	0
Gender	Female	5	9	3
	Male	3	2	1
	Gelding	8	4	5
Age	Min	6	7	8
	Mean (sd)	8.7 (2.5)	9.2 (1.9)	10.4 (1.9)
	Max	15	14	13
Mean speed (km/h)	Min	12.66	15.71	15.43
	Mean (sd)	16.81 (2.02)	17.75 (1.34)	16.67 (1.25)
	Max	19.56	19.77	19.05
HR at finish (bpm)	Min	46	50	45
	Mean (sd)	54.9 (6.4)	55.1 (3.8)	55.8 (5.5)
	Max	64	61	63

### NMR Spectra of Equine Plasma

**Figure 1:**
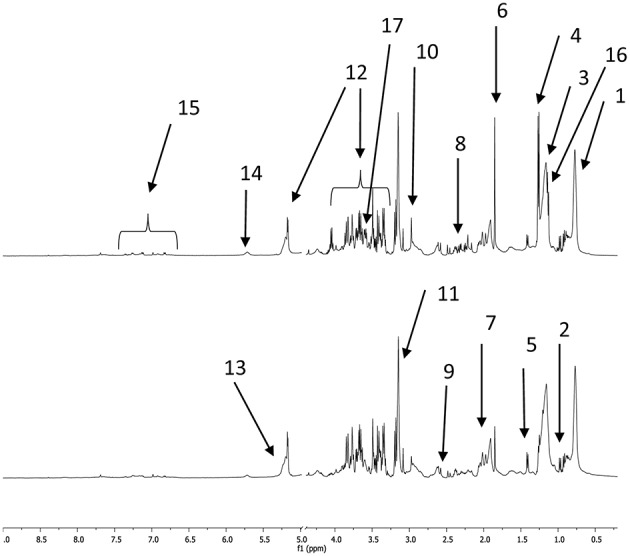
1D proton NMR CPMG spectra of horse plasma samples collected before a 160 km race (bottom spectrum) and afterwards (top spectrum). The main metabolites are labeled as follows (although metabolites that appeared in both spectra are only indicated in one spectrum or the other); 1: methyl moieties from fatty acids, 2: BCAAs (valine, leucine, isoleucine), 3: methylene moieties from fatty acids, 4: lactate, 5: alanine, 6: acetate, 7: N-acetyl moieties 1, 2, and 3, 8: glutamate and glutamine, 9: citrate, 10: creatine, 11: phosphocholine and choline, 12: glucose, 13: alkene moieties from fatty acids, 14: urea, 15: AAAs (tyrosine and phenylalanine), 16: beta hydroxybutyrate, 17: glycerol.

Examples of CPMG spectra obtained with plasma samples taken before and after the 160 km race are shown in Figure 1. Most of the metabolites described below appear in these spectra.

### Metabolomic Profiles in the Three Distance Groups

**Figure 2:**
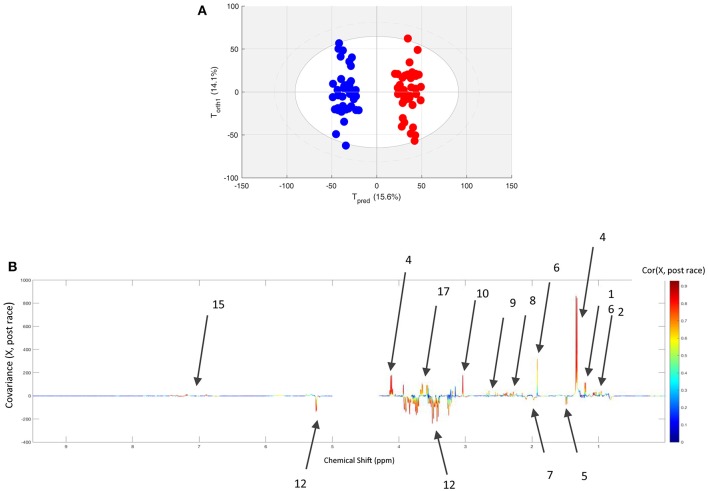
**(A)** The score plot for the O-PLS model computed with paired pre- and post-race plasma samples from all finisher horses. Tpred and Torth correspond to the predictive and orthogonal axes, respectively. Each dot corresponds to a spectrum (in blue for pre-race samples and in red for post-race samples). **(B)** The loading plot for the score plot's predictive axis. The metabolite correlations are represented on a color scale. Positive signals correspond to metabolites present at increased levels in post-race samples. Conversely, negative signals correspond to metabolites present at increased levels at AE. The bins are labeled according to the metabolite assignments in Figure 1.

The spectra of pre- or post-race samples collected from 40 horses having finished one of the three race distances were classified using an O-PLS model. The quality of the fit (as calculated with two components) was high, with R^2^Y = 0.957 and Q^2^Y = 0.924 calculated with 2 components. The score plot and loading plot of this analysis are presented in the Figure 2; the metabolites involved in the discrimination (with an *R*-value over 0.5) are colored from green to red, and are also listed in Table 2. Relative to pre-race samples, the post-race samples contained higher levels of branched-chain amino acids (BCAAs), lactate, acetate, glutamine, glutamate, citrate, creatine, glycerol, and aromatic amino acids (AAAs), and lower levels of glucose, alanine, and compounds including N-acetyl moieties (such as glucosamine in hyaluronic acid).

**Table 2:** Metabolites and their coefficients for the correlation between signal intensity and the race effect for the five calculated O-PLS models.

**Chemical shift**	**Signal assignments**	**All finishers *p* = 10^**−7**^**	**Distance *p* = 10^**−5**^**	**90 km *p* = 10^**−4**^**	**120 km *p* = 10^**−6**^**	**160 km *p* = 10^**−3**^**
0.84	CH_3_ lipids		−0.443		−0.78	−0.613
0.91	Isoleucine			0.696		
0.96	Leucine	0.7		0.792	0.76	0.748
0.98; 1.03	Valine	0.6425		0.7045	0.798	0.6865
1.06	3-Hydroxy isobutyrate	0.883		0.876	0.961	0.966
1.13	Alpha-oxo-isovalerate	0.52		0.67	0.625	0.664
1.19; 4.15	Beta hydroxybutyrate	0.678	0.514	0.701	0.823	0.96
1.23	CH_2_ lipids		−0.429	0.597	0.358	−0.582
1.32;4.10	Lactate	0.8815	−0.42	0.8955	0.9305	0.82
1.47	Alanine	−0.732		−0.629	−0.789	−0.887
1.91	Acetate	0.664	0.552		0.769	0.926
1.96	N-Acetyl 1	−0.694		−0.575	−0.868	−0.776
2.07	N-Acetyl 3	−0.728	−0.527	−0.607	−0.885	−0.768
2.14	Glutamate + glutamine	0.772		0.773	0.783	0.929
2.22	Acetone	0.621				
2.27	Acetoacetate	0.748	0.582	0.587	0.943	0.826
2.33	Glutamate	0.81	0.541	0.711	0.508	0.923
2.41	Glutamine	0.765	0.639	0.74	0.941	0.966
2.51; 2.54 2.64;2.68	Citrate	0.651		0.7625	0.809	
2.97	Lysine					
3.03 3.93	Creatine	0.888	0.583	0.911	0.963	0.915
3.18	Choline	0.697		0.69	0.666	0.891
3.20	Phosphocholine	−0.587		−0.515	−0.901	−0.499
3.24, 3.41–3.91	Glucose	−0.726		−0.8265	−0.877	−0.766
3.57; 3.64; 3.66	Glycerol	0.743		0.68	0.797	0.768
5.23	Glucose	−0.774		−0.821	−0.799	−0.846
5.28	CH = CH lipids		−0.535	0.765		−0.522
5.77	Urea	0.577	0.511	0.507	0.493	0.831
6.89; 7.18	Tyrosine	0.8725		0.904	0.911	0.94
7.04	Histidine					
7.291	Tryptophan	0.666		0.725	0.711	0.851
7.37	Phenylalanine	0.578		0.825	0.693	0.634

*Only metabolites presenting a correlation coefficient over I0.5I are given. The p-value for R = l0.5l is given for each model. All finishers: a comparison of pre- and post-races samples for all 40 horses in the study, regardless of the race distance; distance: a model of post-race samples as a function of the race distance; 90, 120, and 160 km: comparisons of pre- and post-race samples for each race distance separately*.

### Differences in Plasma Metabolic Profiles Between Racing Distance Groups

**Figure 3:**
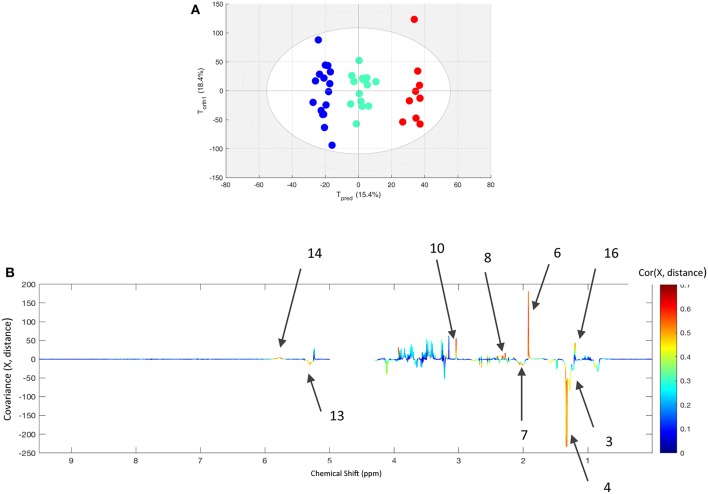
**(A)** The score plot for the best O-PLS model computed with the after exercise samples of all finisher horses with the race distance as supervising factor. Tpred and Torth correspond to the predictive and orthogonal axes, respectively. Each dot corresponds to a spectrum (in blue for 90 km race, green for 120 km race, and red for 160 km race). **(B)** The loading plot for the score plot's predictive axis. The metabolite correlations are represented on a color scale. Positive signals correspond to an increase in the metabolite as the distance increases. Conversely, negative signals correspond to a decrease in the metabolite as the distance increases. The bins are labeled according to the metabolite assignments in Figure 1.

In order to assess the influence of the race distance (90, 120, or 160 km), the post-race data from finishers were extracted from the within-X matrix. This new matrix was used to compute an O-PLS model with distance as the supervising factor (Figure 3). Nine horses (mean age: 10.4 ± 1.9) were included in the analysis of the 160 km race, with 15 (mean age: 9.2 ± 1.9) for the 120 km race, and 16 (mean age: 8.75 ± 2.5) for the 90 km race. The model distinguished between the three distances with an acceptable level of fit (R^2^Y = 0.920 and Q^2^Y = 0.595). The loading plot showed that post-race samples (relative to pre-race samples) contained higher levels of acetate, glutamate, glutamine, citrate, creatine and urea, and lower levels of lactate, lipids, and N-acetyl functional groups when race distance increases.

**Figure 4:**
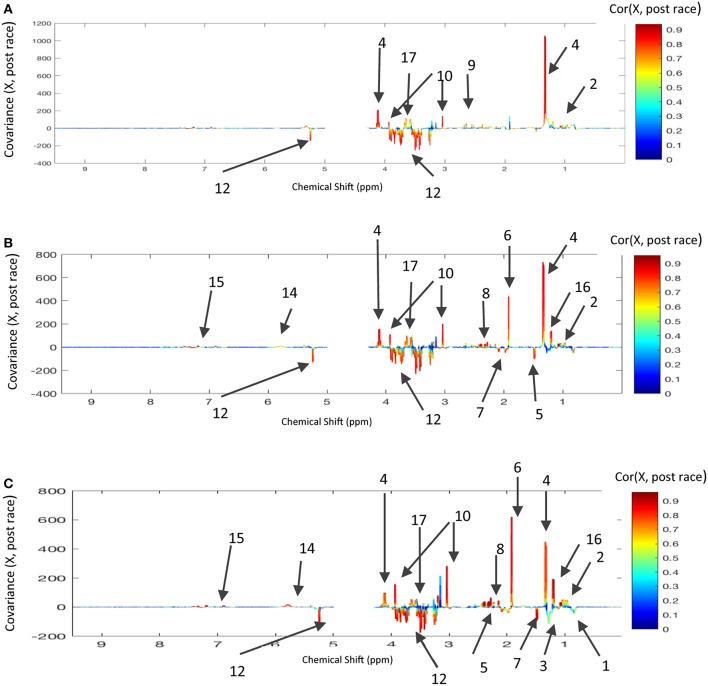
Loading plots for the score plots' predictive axis (not shown) in O-PLS models calculated for pre-race and post-race samples and the three race distances: **(A)** 90 km and 16 horses, three components; R^2^Y = 0.993, Q^2^Y = 0.874; **(B)** 120 km and 15 horses, three components, R^2^Y = 0.986, Q^2^Y = 0.947, **(C)** 160 km and 9 horses, three components, R^2^Y = 0.995, Q^2^Y = 0.839. The metabolite correlations are represented on a color scale. Positive signals correspond to metabolites present at increased levels in post-race samples. Conversely, negative signals correspond to metabolites present at increased levels in post-race samples. The bins are labeled according to the metabolite assignments in Figure 1.

To confirm the metabolic variations as a function of the distance covered, we computed three models comparing pre- vs. post-race NMR data. Again, inter-individual variability was removed by considering only the within-X data. All three models had a good statistical performance, as shown by the loading plots (Figure 4). The discriminant metabolites are reported in Table 2. Glucose, glycerol, BCAAs, and AAAs were discriminant metabolites in all three models, which confirmed the results described above (Figure 3) for the O-PLS model as a function of distance. Relative to pre-race samples, post-race samples had lower levels of lipids and acetate in 120 and 160 km races but not in the 90 km race. Several correlation coefficients (including those for beta-hydroxybutyrate, lactate, creatine, and urea) differed as a function of the race distance (Table 2), which explains why these metabolites were discriminant when modeling the distance with the NMR data from post-race samples.

### Common Analysis of Biochemical Data and NMR Spectra

A non-supervised CCSWA was used to identify the NMR and biochemical assay variables involved in common principal components. Samples from one of the horses in the 120 km event were removed from the analysis because of an out-of-range CK activity (>100,000 UI, i.e., more than a hundred times the upper normal limit).

**Figure 5:**
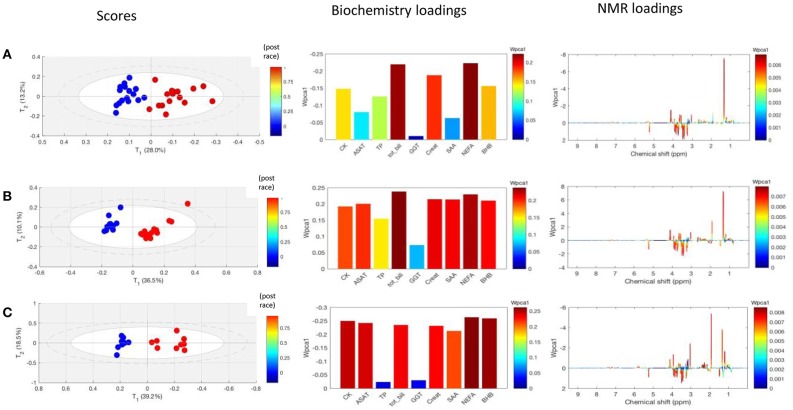
CCSWA models obtained for NMR spectra and biochemical assay data derived from pre-race samples (blue dots) and post-race samples (red dots) for the three race distances. The first column corresponds to the score plots, the second corresponds to the biochemical variables' loadings for the model's first principal component of the model and the third corresponds to the NMR variables' loadings for the first principal component of the model. **(A)** A model for the 90 km race; fraction of the variance in the first principal component: 0.41 for biochemistry and 0.16 for NMR. **(B)** A model for the 120 km race; fraction of the variance in the first principal component: 0.53 for biochemistry and 0.20 for NMR. **(C)** A model for the 160 km; fraction of the variance in the first principal component: 0.58 for biochemistry and 0.19 (NMR). CK, creatine kinase; ASAT, aspartate-aminotransferase, TP, total protein; Tot Bili, total bilirubin; GGT, gamma glutamyl transferase; Creat, creatinine; SAA, serum amyloid A; NEFA, non-esterified fatty acid; BHB, β-hydroxy butyrate.

In an initial model comprising the 40 finishers, the principal component clearly distinguished between pre- and post-race samples. The NMR data and biochemistry data, respectively, accounted for 19 and 58% of the component's variability. Next, CCSWA models were calculated separately for pre- vs. post-race samples from horses in each of the three race distances. The models' principal component distinguished between the three distances (Figure 5). The loading plots showed that the discriminant metabolites in the CCSWA models were the same as those in the O-PLS models (Figure 3). The weights of the biochemistry parameter differed as a function of the distance covered (Table 3). Non-esterified fatty acids had a high weight in all three models, bilirubin and creatinine had a high weight in the 90 and 120 km models, and CK and ASAT activities had a high weight in the 160 km model. Gamma glutamyl transferase made the lowest contribution to the principal component in all three models.

**Table 3:** Ranking of the contribution of biochemical variables to principal component in the CCSWA, for each race distance.

**Rank**	**90 km**	**120 km**	**160 km**
*1*	NEFAs	Total bili	NEFA
*2*	Total bili	NEFA	BHB
*3*	Creatinine	Creatinine	CK
*4*	BHB	SAA	ASAT
*5*	CK	BHB	Total bili
*6*	TP	ASAT	Creatinine
*7*	ASAT	CK	SAA
*8*	SAA	TP	TP
*9*	GGT	GGT	GGT

*CK, creatine kinase; ASAT, aspartate-aminotransferase; TP, total protein; Tot Bili, total bilirubin; GGT, gamma glutamyl transferase; SAA, serum amyloid A; NEFAs, non-esterified fatty acids; BHB, beta-hydroxybutyrate*.

## Discussion

Our findings on the effect of equine endurance racing on the plasma metabolome are in line with previous studies (Le Moyec et al., 2014; Luck et al., 2015). However, our experimental design enabled us to highlight the effects of distance. All the horses had traveled to arrive at the competition site between 4 and 36 h before sampling. At the time of the race (in October), the weather was cool and favorable for good conditions of transport. However, the individual conditions and the duration of transport are not known nor standardized. It is well known that transport generates stress that can have consequences on the health of horses all the more marked as the duration increases and even modify the microbiota (Perry et al., 2018). It is likely that pre-race measurements differ from what would have been obtained in non-transported horses. This did not seem to be too limiting as all the horses were transported and we evaluated the effects of the race distance by comparing the measurements before and after the race.

In contrast to earlier studies, pre- and post-race samples were collected from all the study participants—making it possible to perform the metabolomic analysis on paired samples and thus to eliminate inter-individual variability. Of the 52 horses initially sampled, 40 finished the 90, 120, or 160 km race under the same environmental conditions; this enabled us to investigate the influence of race distance on metabolic adaptations. Of course, it would have been better to sample the horses at each vet-gate and compare the groups of horses at the same distance. However, while most riders agreed their horses being sampled before and after the race, the horses must rest, drink and eat at each halt between the loops. Performing blood sampling at this time disturbs them; the risk of creating a hematoma at the sampling site is high. In addition, the rules of the competition don't allow collecting blood samples during the race. In the present study, we asked before the completion, the authorization of the judges for blood sampling before the start and after finish of the race. Conversely, usual race strategy is that riders retain their horses on the first loops of the race and run the last loop to the maximum of the possibilities of their horse. Sampling horses running 160 km at 90 km is not the same as sampling horses that have just finished a 90 km race. Another limitation is differences in training status of horses. The horses in the three races did not differ greatly with regard to age or breed. Heart rate and speed data during the same part of the race were also the same between the three distance groups. All horses in the study had several years of experience in endurance racing. However, we cannot say that the groups were equal, since very few 90 km race horses had already run 120 km and even less 160 km. Conversely, all the 160 km-horses had experience over 120 and 90 km races. Experience and level of training can therefore be confounders in our study. The metabolites found discriminant in our multivariate statistical models as collected in the Table 2 do not represent an exhaustive list of metabolites that could be detected in the plasma NMR spectra but only those found correlated to the race status (pre- or post- race). Obviously, other chemical compounds may be identified in horses' plasma using NMR or mass spectrometry (Escalona et al., 2015). Nevertheless, the output of the O-PLS model calculated with paired data showed that the three main metabolic pathways (carbohydrate, protein, and lipid pathways) were all altered by the race. The biochemistry investigation performed in parallel showed that the parameters were also affected after the race. Depending on the race distance common variability could be found between both types of data, biochemistry and NMR.

Endurance races place high demands on energy metabolism (Barrey, 1993) which takes place mainly within the striated muscles and liver. The high-energy demand provoked by endurance exercise causes disruption in plasma biochemistry homeostasis. Our OPLS model showed that the horses' glycaemia was lower in post-race plasma samples than in pre-race samples. Glucose seems to contribute to a lesser extend for the 160 km race; since the fall in glycaemia was smaller for the longest distance. Several explanations can be put forward to explain a lesser glucose level decrease in 160 km horses. These horses may better compensate for glucose losses through a better use of muscle and liver glycogenolysis or a lack of necessary gluconeogenesis. They are probably better trained in this long race category and thus, could increase their muscular and liver glycogene reserves. However, other metabolic changes observed in parallel lead us to believe that horses of 160 km preferentially use the metabolism of fatty acids for muscular functioning and thus save their glucose and avoid fatigue. In all horses, the plasma lactate level was significantly increased after the race. One can therefore hypothesize that glucose was anaerobically metabolized at least during the last minutes of exercise when the riders asked their horses to increase their speed to the finish line. Lactate was a discriminant metabolite in the model comparing the three distances. The increase in lactate level was smaller for the 160 km race—showing that oxidative metabolic pathways were more active over the longest distance. Changes in lactate and glucose content during exercise have been extensively investigated; the lactate content was found not to depend on the horses' age (Kang and Park, 2017) or the racing distance (Cywińska et al., 2012). However, Cywińska et al. studied much shorter distances (34 and 60 km) than those assessed here (90, 120, and 160 km). In addition, the blood lactate increase is probably more related to the faster gallop asked by the riders the last minutes on the finish line than to the slow regular canter maintained during most of the race distance. As shown by the average speeds during the last loop, in the 90 km race, the capacity to increase the speed at finish was higher than in the 160 km race, thus higher blood lactate is expected at the arrival of 90 km race. Taken as a whole, the data on glucose and lactate levels vs. race distance suggest that compensatory metabolic pathways only replace anaerobic glycolysis after a certain time interval. This time interval depends on the race distance, the rider's race strategy as well as on the training status of the horse.

Metabolism of the amino acid alanine is linked to the glycolysis pathway; hence, the observed decrease in alanine levels during all three races can be explained by transformation of this amino acid into pyruvate, in order to maintain a sufficient level of substrate for the tricarboxylic acid (TCA) cycle despite a decrease in the glucose level. However, the decrease in the alanine level did not vary as a function of the distance covered. Variations in the citrate level are also of interest in this respect. Citrate is one of the tricarboxylic acids in the TCA cycle, and citrate synthase is frequently used as a marker of mitochondrial activity (Rasmussen et al., 2001). When considering horses having finished the 90 km race or the 120 km race, post-race samples contained higher citrate levels than pre-race samples. This modulation is evidenced in the plasma compartment and may be difficult to rely to mitochondrial citrate content (Votion et al., 2010), which is also dependent on the aconitase activity. However, citrate was not a discriminant metabolite for the 160 km race—suggesting the involvement of a different mechanism in horses covering this distance.

Physiological and metabolic adaptations may also involve lipid oxidation. Lipid metabolism is known to take place during endurance exercise, as a mean of maintaining the energy supply as the glucose level falls (Barrey, 1993). Energy supply by fatty acid breakdown is an oxidative mechanism that produces two carbon residues for entry into the TCA cycle as acetyl-CoA. The oxidation of fatty acid chains results in the release of ketone bodies such as acetate, acetoacetate, and hydroxybutyrate. The oxidized fatty acids come from the hydrolysis of triglycerides by lipases. Similarly, the hydrolysis of phospholipids produces two fatty acids and a glycerophosphocholine or glycerophosphoserine residue. Choline-containing phospholipids are the most abundant lipids within the cell membrane. Glycerophosphocholine is further hydrolyzed into glycerol, and phosphocholine is further hydrolyzed into choline and a phosphate residue.

According to the O-PLS model computed with the whole set of samples, lipid consumption is taking place as several ketone bodies are detected, with glycerol and choline increases after the race. However, the lipid signals (including methyl and methylene signals at 0.8 and 1.20 ppm and double bounds at 5.3 ppm) are not participating to the model including all horses. As the levels of circulating lipids included in lipoprotein particles do not change, it might be that the lipids entering this energy supply pathway are taken from another pool of lipids. Several sources of lipids can provide triglycerides including adipocytes and most likely the muscle tissue itself. On the other hand, lipid signals (mainly fatty acids from lipoproteins) were discriminant in the O-PLS model computed with the samples from all three distances. This finding was confirmed by the models computed for the 160 km distance, in which lipids discriminated between the pre- and post-race samples. However, this was not the case for the 90 and 120 km races. The elevated level of lipid consumption was confirmed by the greater release of ketone bodies detectable in NMR spectra and confirmed in the CCSWA model by the BHB weight. The acetate level was not discriminant in the 90 km model but was elevated in the 120 and 160 km races. Acetate was also a discriminant metabolite in the model calculated for the post-race samples at all three distances. The initiation of lipid oxidation might require greater adaptation—such as that obtained through training, for example. Longer race duration might also trigger this initiation as a means of compensating for greater glucose consumption. We also found that the level of NEFAs was higher in post-race samples. In the CCSWA model calculated with both biochemistry and NMR data, NEFAs had the greatest weight at each of the three distances.

In view of the need for energy production, protein metabolism was also affected by the endurance racing. This is demonstrated by the loading plot for the O-PLS model calculated with NMR data from all three distances. We observed pre-/post-race differences in the levels of several amino acids. With the exception of alanine (discussed above), the levels of all other detected amino acids were higher in post-race samples than in pre-race samples. They included BCAAs (namely leucine and valine), glutamate, glutamine, and AAAs (namely tyrosine and phenylalanine). This increase in amino acid plasma content might be due to proteolysis within muscle cells (Xu et al., 2017). This proteolysis contributes to energy supply, since amino acids can enter the TCA cycle after deamination. The presence of the BCAA metabolite oxo-isovalerate in post-race samples confirms the occurrence of proteolysis at the end of the event.

When the effects of distance on post-race samples were compared, the glutamine and glutamate contributed to the model but the BCAAs leucine and valine and the AAAs tyrosine and phenylalanine did not. Given that glutamate enters the TCA cycle after a single enzymatic reaction, we hypothesize that levels of glutamate deamination are lower in the 90 km race than in the 120 and 160 km races. In contrast, the release of BCAAs and AAAs into the plasma was similar after the three distances. Moreover, the fact that the level of the valine metabolite oxo-isovalerate was higher in post-race samples than in pre-race samples suggested that valine had been released by proteolysis and catabolized.

Elevated creatine level and CK activity are also associated with greater race distances, suggesting that long and/or intense exercise induce a higher membrane permeability associated to leakage of muscle proteins in blood (Valberg et al., 1993; Barrey et al., 2010; Serteyn et al., 2010). The increase of CK activity in the blood is associated with a severe inflammation signal according to the blood transcriptome during the race and may reach muscular damage and rhabdomyolysis (Barrey et al., 2010; Capomaccio et al., 2010; Mach et al., 2016). Tryptophan and histidine are not modified for the three distances while tyrosine and phenylalanine are present in higher amount in plasma collected after races than those collected before races. The tryptophan:tyrosine ratio after exercise has been extensively investigated because these metabolites are involved in inflammatory processes and in the metabolism of the neurotransmitter serotonin (Strasser et al., 2016). It is difficult to conclude as to the presence of inflammation on the basis of the NMR data alone; moreover, tryptophan levels did not appear to vary as a function of the race distance. Phenylalanine and tyrosine levels changed in the same way as the BCAAs; levels were higher after the race but (in contrast to glutamine and glutamate) did not vary as a function of the race distance. Overall, one can conclude that (i) proteolysis occurs during endurance races and (ii) glutamine and glutamate contribute to the increase in energy supply as the race distance rises.

Unlike the proton NMR spectra of human plasma, spectra of horse plasma contain three resonances at around 2.1 ppm; we and others have attributed them non-specifically to the N-acetyl moieties of glycoproteins (Hodavance et al., 2007; Luck et al., 2015). We hypothesize that the resonances correspond to hyaluronic acid metabolism and turnover during an endurance effort. Furthermore, the resonances were not assigned in Escalona et al.'s recent assignment of most of the metabolites in equine biofluids detected by NMR spectroscopy (Escalona et al., 2015). Nevertheless, the signals' chemical shifts suggest that they are produced by N-acetyl moieties of polymeric molecules, such as glucosamine. The observed decrease in the intensity of two of these signals after the endurance races suggests that N-acetyl moieties are consumed or catabolized during intense exercise.

## Conclusion

Using NMR metabolomics, we confirmed that 90, 120, and 160 km races had a marked impact on the metabolic pathways involved in energy supply in endurance horses. The experimental design enabled to evidence a metabolic adaptation of the horses associated to the total race distance without identifying the origin. The factors may be diverse and interacting: exercise distance and duration, rider's management of the race, training status of the horse, and inherited endurance capacity of the horse. However, we evidenced an adaptive metabolic switch toward lipid metabolism to progress from the shortest race distance (90 km) to the highest race distance (160 km). It might be possible to promote the use of lipid metabolism through appropriate training, dietary measures, and race tactics. More research is needed to understand metabolic shifts that take place in horses throughout different types of exercise.

## Ethics Statement

ComEth EnvA-Upec-ANSES; reference: 11-0041, dated July 12th 2011.

## Author Contributions

LL, EB, and CR conceived and designed the experiments. CR, NM, EB, LL, MT, NB, JR, and EZ-R performed the experiments. LL, EB, CR, and MT analyzed the data.

### Conflict of Interest Statement

The authors declare that the research was conducted in the absence of any commercial or financial relationships that could be construed as a potential conflict of interest.
