# Supplementary material for: A First Step Toward Unraveling the Energy Metabolism in Endurance Horses: Comparison of Plasma Nuclear Magnetic Resonance Metabolomic Profiles Before and After Different Endurance Race Distances
Source: Front Mol Biosci. 2019 Jun 12;6:45. doi: 10.3389/fmolb.2019.00045 (PMC6581711; doi:10.3389/fmolb.2019.00045)
Supplement: Supplementary file 1 [file Table_1.docx]

Supplemental Table S1: Time of start, phase length and hold time duration for each race.

|  | CEI*** |  | CEI** |  | CEI* |  |
| --- | --- | --- | --- | --- | --- | --- |
| Time of start | 06:00 |  | 07:45 |  | 08:15 |  |
| Phase | km | Hold time (minutes) | km | Hold time (minutes) | km | Hold time (minutes) |
| 1 | 34 | 40 | 34 | 40 | 36 | 40 |
| 2 | 24 | 40 | 36 | 40 | 30 | 50 |
| 3 | 36 | 40 | 30 | 50 | 23 |  |
| 4 | 23 | 50 | 20 |  |  |  |
| 5 | 20 | 50 |  |  |  |  |
| 6 | 23 |  |  |  |  |  |
| Total | 160 | 220 | 120 | 130 | 89 | 90 |
| Arrivals | 18:03:55 | 20:02:18 | 16:09:17 | 17:33:24 | 14:18:02 | 16:46:39 |
